# Ecological factors associated with child sexual abuse among 15- to 17-year-old adolescents in mainland China: implications for intervention

**DOI:** 10.3389/fpubh.2023.1169669

**Published:** 2023-10-20

**Authors:** Guochen Fu, Yao Xu, Mingliang Pan, Ziyuan Zhang, Hudie Zhang, Youxiong Zhao, Lu Lin, Zijie Ye, Jiajun Liu, Fangjun Lan, Dongsheng Luo, Siyi Wang, Bangzheng Zhu, Xinyu Liao, Mengsi Hong, Jilun Chen, Zihao Li, Gaoming Yang, Ziyuan Zhao, Yusi Liu, Fang Ruan, Chunyan Yang, Junfang Wang

**Affiliations:** Department of Preventive Medicine, Hubei University of Science and Technology, Xianning, China

**Keywords:** child sexual abuse, adolescents, gender, sexual minority, ecological model

## Abstract

**Background:**

Child sexual abuse is a major public health problem with adverse consequences for victims’ physical, mental, and reproductive health. This cross-sectional study aimed to determine the prevalence of child sexual abuse and its associated factors among 15- to 17-year-old adolescents in mainland China.

**Methods:**

From September 8, 2019 to January 17, 2020, a total of 48,660 participants were recruited by 58 colleges and universities across the whole country to complete the self-administered, structured, online questionnaire. This analysis was restricted to 3,215 adolescents aged between 15 and 17 years in mainland China. Chi-square tests and multivariate Logistic regression analyses were performed to identify individual, relationship, and community factors associated with child sexual abuse.

**Results:**

The overall prevalence of child sexual abuse was 12.0%. More specifically, 13.0% of girls and 10.6% of boys reported that they were sexually abused prior to 18 years of age. At the individual level, being female, sexual minority identity, younger age, and higher levels of knowledge, skills and self-efficacy regarding condom use were significantly related to increased odds of reporting sexual abuse. At the relationship and community level, adolescents from disrupted families and those entering into a marriage, having casual sexual partners, and having first intercourse at a younger age were more likely to report sexual abuse. On the contrary, those who had never discussed sex-related topics with their family members at home and were offered school-based sexuality education later (vs. earlier) were less likely to report sexual abuse.

**Conclusion:**

Multilevel prevention programs and strategies, including targeting adolescents with high-risk characteristics, educating young children and their parents about child sexual abuse prevention and optimizing the involvement of parents, school, community, society and government in comprehensive sexuality education, should be taken to reduce child sexual abuse among 15- to 17-year-old adolescents.

## Background

Child sexual abuse is typically defined as the involvement of children in “any sexual act, attempt to obtain a sexual act, unwanted sexual comments or advances or acts to traffic, or otherwise directed, against a person’s sexuality using coercion, by any person regardless of their relationship to the victim, in any setting, including but not limited to home and work” (p. 149) (1). Broadly, it includes three forms (2), ranging from unwanted verbal sexual propositions or harassment (i.e., non-contact sexual abuse), to unwanted touching of the sexual organs, breast and buttocks (i.e., contact sexual abuse without penetration), to unwanted oral sex, anal or vaginal intercourse (i.e., penetrative sexual abuse). Child sexual abuse has been well documented to be associated with a wide range of physical, social and psychological health adverse consequences, including but not limited to physical injury, sexually transmitted infections, sexual functioning issues, risk-taking behaviors, post-traumatic stress disorder and attempted suicide (1, 3–8).

Child sexual abuse is a major public health problem affecting people of virtually all ages, genders and socioeconomic backgrounds in both developed and developing countries (9–26). Child sexual abuse among minors in mainland China has become the focus of recent research (19–26). The relatively high prevalence of child sexual abuse and the newly passed law that criminalizes raping and molesting the minors and protects the rights and interests of the minors makes this focus understandable. For example, a meta-analysis conducted by Peng et al. (20) indicated that China had a higher combined incidence rate of child sexual abuse (18.2%) than the international average level of 11.8% (15). The existing studies also attempted to explore the associations of individual [e.g., gender (19, 26), sexual orientation (3) and dating behaviors (23)], family [e.g., parenting style (23) and parental education (21, 26)], and community factors [e.g., rural/urban residence (19, 26) and geographical characteristics (19)] with child sexual abuse. However, these studies have typically employed samples of students selected from metropolitan cities such as Guangzhou (23) and Hefei (3), or/and from several provinces (22, 26) and seldom systematically examined the effects of individual, relationship, community and societal attitudes on child sexual abuse. Therefore, the present study drew a large, diverse sample of adolescents from across the whole country to assess the prevalence of child sexual abuse and fully analyze its associated factors, so that proper measures can be taken to prevent the occurrence of this phenomenon.

## Theoretical framework

According to the social-ecological model, factors associated with sexual abuse can be divided into four levels (27): individual, relationship, community, and society. Briefly, individual-level factors, representing the most proximate predictors of sexual abuse, include nonmodifiable attributes [e.g., gender (19, 27), sexual orientation (3), age (19)] and modifiable factors [i.e., sexual attitudes and cognitions (28)].

At the relationship level, sexual violence has been found to be associated with the influence of parents (2, 23, 27, 29), peers or partners (1, 2, 27, 28) and spouses (30). For example, according to study conducted by Priebe and Svedin (2), students with a history of child sexual abuse were less likely to be live with both parents. Similarly, Bhochhibhoya et al. (27) reported that more than one-third of college students were exposed to sexual violence by an intimate partner. In addition, Arata and Lindman (30) found that dating behaviors (particularly the number of sexual and/or dating partners) and marital status were related to child sexual abuse and revictimization experience. Sex education including parent–child sex communication at home (29) and school-based sex education (31), was found to be related to sexual behaviors, but there is not yet empirical evidence indicating its role in prevention of sexual abuse.

At the community and societal level, the geographical origin of the sample was found to be associated with child sexual abuse in a meta-analysis conducted by Stoltenborgh et al. (15). More specifically, the Asian continent including China (32) had the lowest prevalence of child sexual abuse. Furthermore, Ji et al. (19) found that children from urban areas and non-mainland areas in China were less likely to be victims of sexual abuse. In addition, perceived discrimination has been found to be significantly associated with sexual victimization among sexual minority populations (33) and among college students in dating relationships (27).

## Hypotheses

**Figure 1:**
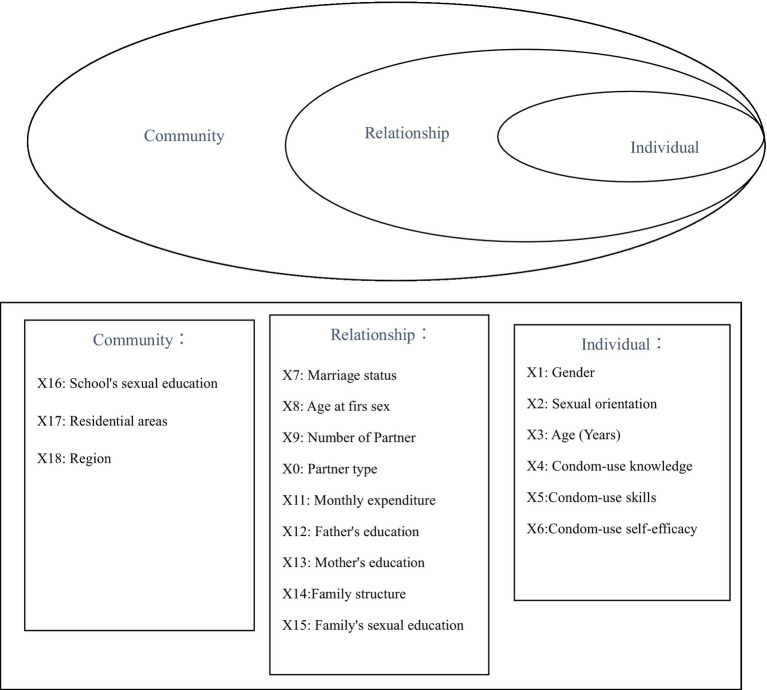
Determinants of child sexual abuse among 15- to 17-year-old adolescents based on the ecological model. Adapted from Bhochhibhoya et al. (27).

Therefore, guided by the socio-ecological model and based on the findings from previous studies, 18 variables hypothesized to influence child sexual abuse were categorized into individual, relationship and community-level as indicated in Figure 1. It was hypothesized that at the individual level, females and sexual minority adolescents would be more likely to report sexual abuse. It was also hypothesized that adolescents from disrupted families and those who had casual sexual partners, entered into a marriage, and had first intercourse at a younger age were more likely to experience sexual abuse. The role of other individual factors (i.e., age, condom-use knowledge, skills and self-efficacy), family-based and school-based sex education, regions and area of residence was examined in a more exploratory fashion.

## Methods

### Study design and setting

The data used in this paper were taken from a web-based cross-sectional survey on knowledge, attitude and risk behavior about reproductive health and sex health education, carried out from September 8, 2019 to January 17, 2020. Both convenience sampling and snowball sampling methods were applied to recruit participants. The detailed description of sampling methods has been presented in our previous papers (34–36). Briefly, due to their convenience, participants currently enrolled in courses offered by Hubei University of Science and Technology (HUSC) were first invited to complete the online questionnaire in their classroom during a regular class period. Meanwhile, a series of incentive measures were adopted to recruit more participants, such as earning one extra credit point or being paid 50 cents in RMB for each completed questionnaire. In order to obtain a large national sample, our research team also distributed the URLs of this survey in various ways such as via Wechat and Sina Weibo (the largest social media platforms in mainland China) to potential participants, while cooperating with 57 other colleges and universities. Once colleges and universities agreed to cooperate with us to participate in the survey, we would send them a questionnaire link and their respective barcodes. All participants would answer the questionnaire by scanning the two dimensional barcodes of the questionnaire address or clicking the relevant link.

### Participants

The survey involved a total of 48,660 participants distributed across the whole country. All statistical analyses in this study were confined to 3,215 adolescents, who must meet the following three inclusion criteria: (a) aged 15–17 years; (b) currently living in the Chinese mainland [Participants from abroad and non-mainland areas (i.e., Hong Kang, Taiwan, and Macao)] were excluded from the present analysis due to small sample size; (c) answering the questionnaire no later than January 17, 2020.

### Ethics

The content and purpose of the study was explained to each subject in advance and electronic informed consent was obtained from all participants enrolled in the study. Participants were also guaranteed that they could withdraw from the survey at any time. This study received ethical approval (No. 2021XG001) from the academic ethics and moral supervision committee of HUSC. Prior to conducting the survey, an official approval was also obtained from the Director of Students’ Affairs Division of HUSC and 57 other colleges and universities.

### Design and content of the questionnaire

The questionnaire was developed based on the ecological model and the existing literature on sexual abuse and was reviewed by experts on this topic. The instrument was then pilot tested with 50 students from HUSC.

### Dependent variable

The dependent variable in this study was the history of child sexual abuse, which was measured by directly asking the participant, “Have you ever experienced sexual abuse?” (37) (“你曾经遭受过性侵犯吗?”). Participants who responded “Yes” were classified as the exposed group and codes as 1, and those who answered “No” or “Not sure” were classified as the non-exposed group and codes as 0.

### Independent variables

As indicated in Figure 1, 18 independent variables were grouped into three levels (individual, relationship, and community) based on the ecological model.

### The individual factors

The individual variables included in this study were demographic information of the participant (i.e., gender, sexual orientation, and age), condom use knowledge, skills and self-efficacy. Consistent with other studies (38, 39), gender was dichotomised into male and female, and sexual orientation was categorized into two groups (sexual minority and heterosexual). The sample was limited to adolescents aged 15–17 years to assess sexual abuse prior to 18 years of age (i.e., child sexual abuse), because the age of onset of sexual abuse was not collected in the questionnaire.

Condom use knowledge was measured by asking the participants whether they agreed with the statement that correct and consistent use of condoms provides dual protection against both unplanned pregnancies and HIV/STI infection, while skills were assessed by asking whether they knew how to use a condom correctly. Individuals who answered “Yes” to these two questions were categorized as having correct knowledge and skills, while those who answered “No” or “Not sure” were classified as having incorrect knowledge or lack of condom-use skills. Condom use self-efficacy was measured by asking the participants to rate their confidence in their ability to negotiate condom use with any sexual partners under any circumstances on a five-point Likert scale ranging from 1 (very confident) to 5 (very unconfident). Undergraduates who circled “very confident” were categorized as having high self-efficacy, while their counterparts (i.e., those who circled “confident,” “not sure,” “unconfident” or “very unconfident”) were classified as having low self-efficacy.

### Relationship, school and community characteristics

The relationship, family, school and community characteristics included marital status, sexual experience and age of sexual initiation, the household income, both paternal and maternal education status, family structure, family-based and school-based sex education, region and area of residence. In order to assess marital status, participants were asked what their current marital status was and five options were provided: single, married, separated, divorced, or widowed. In the final analysis, marital status was categorized into two groups: 0 = Never-married (i.e., single) and 1 = Ever-married (i.e., married, separated, divorced, or widowed).

Participants were first asked to indicate whether they had ever engaged in sexual intercourse. Those who were sexually active were further asked to provide age at first sex, the number of partners and the types of partnership. Consistent with our previous study (40), early sexual debut was defined as having first sexual intercourse before the age of 14 years, the number of partners was dichotomised into single partner (having only one or no sexual partner in the past 6 months) or multiple partners, and partner type was classified into two groups: regular sex (defined as boyfriend/girlfriend) and casual sex (including commercial sex worker and one night stand).

The household income was measured based on the monthly expenditure of a college student and was classified into low- (less than 1,000 Yuan) and high-income groups (≥1,000). Respondents were asked about the highest level of formal schooling their parents had completed using a seven-point response option (1 = “Illiterate,” 2 = “Primary,” 3 = “Middle school,” 4 = “High school,” 5 = “three-year college degree,” 6 = “four- or five-year bachelor degree,” and 7 = “Post-graduate study”). In the final analysis, both paternal and maternal education status was grouped into two categories: Low (Middle school or below) and High (High school or above). Family structure was categorized into intact family (i.e., living with both parents at home) and disrupted family (i.e., living with only one parent or living without parents at home). Furthermore, students were asked whether they had ever discussed sex-related issues with their family members at home and when they receive their formal sex education (i.e., curriculum-based programs). Those who answered “yes” to the former question were classified as receiving sex education at home, and those who were taught sex in childcare settings, preschools or elementary schools were categorized as earlier initiation of sex education. China was geographically divided into three major parts (1 = East, 2 = Middle, 3 = West), and residence was grouped into urban and rural areas.

### Statistical analyses

Ecological factors associated with child sexual abuse were evaluated based on the following four steps. In the first stage, a detailed description was made of all the variables in the socio-ecological model. Second, Pearson’s Chi-square tests were performed to compare differences between those who experienced sexual abuse and those who did not. Third, multicollinearity was checked using the correlation matrix of independent variables and the variance inflation factor (VIF) and tolerance (TOL, defined as the reciprocal of VIF). A general rule of thumb is that the Pearson’s correlation coefficients are below 0.6 and all VIF values are lower than 10 (i.e., all TOL values are greater than 0.1). Finally, a multivariable backward stepwise logistic regression was used to assess the strength of an association between the dependent and independent variables. Only variables with *p* values less than 0.05 were retained in the final model. The adjusted odds ratios (AORs) and their respective 95% confidence intervals (CI) were also reported. All statistical analyses were performed using IBM SPSS version 28.0 Software.

## Results

### Descriptive statistics

Descriptive statistics for the dependent and independent variables of this study are presented in Table 1. As indicated in Table 1, the overall prevalence of child sexual abuse was 12.0% (386/3,215) out of the 3,215 eligible adolescents. More specifically, 13.0% (247/1,899) of girls and 10.6% (139/1,316) of boys reported that they were sexually abused during childhood.

**Table 1:** Individual, interrelation and community characteristics of the 3,215 adolescents aged between 15 and 17 years in mainland China.

Variable	Total (*n* = 3,215)		Ever (*n* = 386)		Never (*n* = 2,829)		*χ* ^2^	*p*
	*n*	%	*n*	%	*n*	%		
X1: Gender								
0 = Male	1,316	40.9	139	36.0	1,177	41.6	4.40	0.036
1 = Female	1899	59.1	247	64.0	1,652	58.4		
X2:Sexual orientation								
0 = Heterosexual	2,736	85.1	248	64.2	2,488	87.9	150.44	<0.001
1 = Sexual minority	479	14.9	138	35.8	341	12.1		
X3: Age (Mean = 16.8 years)								
1 = 15	166	5.2	64	16.6	102	3.6	129.63	<0.001
2 = 16	377	11.7	61	15.8	316	11.2		
3 = 17	2,672	83.1	261	67.6	2,411	85.2		
X4: Condom use knowledge								
0 = Low	1,639	51.0	161	41.7	1,478	52.2	15.08	<0.001
1 = High	1,576	49.0	225	58.3	1,351	47.8		
X5:Condom-use skills								
0 = No	2,715	84.4	298	77.2	2,417	85.4	17.54	<0.001
1 = Yes	500	15.6	88	22.8	412	14.6		
X6:Condom-use self-efficacy								
0 = Low	2,467	76.7	298	64.2	2,417	78.4	38.30	<0.001
1 = High	748	23.3	88	35.8	412	21.6		
X7: Marriage status								
0 = Never-married	3,068	95.4	325	84.2	2,743	97.0	126.81	<0.001
1 = Ever-married	147	4.6	61	15.8	86	3.0		
X8: Age at firs intercourse								
0 = Older than 14	3,138	97.6	327	84.7	2,811	99.4	311.79	<0.001
1 = Younger than 14	77	2.4	59	15.3	18	0.6		
X9: Number of Partner								
0 = Single	3,037	94.5	327	84.7	2,710	95.8	79.71	<0.001
1 = Multiple	178	5.5	59	15.3	119	4.2		
X10: Partner type								
0 = Stable	3,156	98.2	352	91.2	2,804	99.1	118.40	<0.001
1 = Casual	59	1.8	34	8.8	25	0.9		
X11: Monthly expenditure (Yuan)								
0 = <1,000	1,004	31.2	142	36.8	862	30.5	6.31	0.012
1 = ≥1,000	2,211	68.8	244	63.2	1967	69.5		
X12:Father’s education								
0 = Middle school or less	1932	60.1	233	60.4	1,699	60.1	0.01	0.908
1 = High school or more	1,283	39.9	153	39.6	1,130	39.9		
X13:Mother’s education								
0 = Middle school or less	2,163	67.3	255	66.1	1908	67.4	0.30	0.587
1 = High school or more	1,052	32.7	131	33.9	921	32.6		
X14:Family structure								
0 = Intact families	2011	62.6	177	45.9	1834	64.8	52.20	<0.001
1 = Disrupted families	1,204	37.4	209	54.1	995	35.2		
X15:Family’s sexual education								
0 = Yes	718	22.3	144	37.3	574	20.3	56.70	<0.001
1 = No	2,497	77.7	242	62.7	2,255	79.7		
X16: School’s sexual education								
0 = Kindergarden or elementary school	636	19.8	142	36.8	494	17.5	80.46	<0.001
1 = Junior or senior middle school	2,376	73.9	228	59.1	2,148	75.9		
2 = College	203	6.3	16	4.1	187	6.6		
X17: Residential areas								
0 = Rural	1708	53.1	175	45.3	1,533	54.2	10.69	0.001
1 = Urban	1,507	46.9	211	54.7	1,296	45.8		
X18: Region								
1 = East	703	21.9	70	18.1	633	22.4	4.84	0.089
2 = Middle	1791	55.7	217	56.2	1,574	55.6		
3 = West	721	22.4	99	25.6	622	22.0		

Nearly three-fifths (59.1%) were female and 14.9% identified as sexual minority adolescents. Respondents ranged in age from 15 to 17 years, with a mean age of 16.8 years. The majority of adolescents (83.1%) were at the age of 17 years. Although 49.0% agreed that correct and consistent use of condoms can offer dual protection against HIV infection and unwanted pregnancies, only 15.6% reported that they knew how to use condoms correctly and 23.3% felt wholly confident in negotiating condom use with any sexual partners under any circumstance. More than one-tenth (12.4%) of adolescents were sexually active, 4.6% already entered into the marriage relationship, 5.5% admitted to having multiple sexual partners in the past 6 months, 2.4% started their first sex under 14 years of age and even 0.9% had sex with casual partners.

Nearly one-third (31.2%) of adolescents reported that their monthly expenditure was below 1,000 Yuan. A higher percentage of adolescents reported their father (39.9%) completed at least high school in comparison to their mother (32.7%). Slightly less than two thirds (62.6%) came from intact families (i.e., lived with both parents at home), 22.3% reported that they had ever discussed sex-related topics with their family members at home, and even 19.8% received school-based sexuality education in childcare settings, preschools or elementary schools. Slightly more than one-half (53.1%) of adolescents resided in rural areas. The sampled adolescents were unevenly distributed across 31 provinces, municipalities and autonomous regions in mainland China were primarily recruited from the middle of China (55.7%) (Table 1).

### Pearson’s Chi-square tests

Table 1 presented the associations between the history of child sexual abuse and individual, interrelation and community characteristics. Except for parents’ education level and residential regions, all other 15 variables were significantly associated with the history of child sexual abuse (i.e., *p* ≤ 0.05).

### Multicollinearity diagnosis

Table 2 presented the Pearson’s correlation coefficient matrix between the eighteen independent variables. The highest correlation coefficient (0.48) existing between Father’s (X12) and Mother’s education (X13) was less than 0.60 and hence there was no issue of collinearity. As can be seen from Table 3, all VIF values ranged between 1.05 and 1.38, below the acceptability threshold of 10, indicating the absence of multicollinearity. Therefore, no variable was excluded from the multiple logistic regression analysis due to violation of the principle of multicollinearity.

**Table 2:** The matrix of Pearson correlation coefficients of factors associated with child sexual abuse (Y).

	X1	X2	X3	X4	X5	X6	X7	X8	X9	X10	X11	X12	X13	X14	X15	X16	X17	X18
X1	–																	
X2	0.07^***^	–																
X3	0.06^**^	−0.14^***^	–															
X4	−0.18^***^	−0.01	−0.06^**^	–														
X5	−0.23^***^	0.05^*^	−0.03	0.19^***^	–													
X6	−0.19^***^	0.07^***^	−0.07^***^	0.18^***^	0.24^***^	–												
X7	−0.04^**^	0.19^***^	−0.23^***^	0.00	0.07^***^	0.04^*^	–											
X8	−0.09^***^	0.13^***^	−0.28^***^	0.10^***^	0.05^*^	0.15^***^	0.25^***^	–										
X9	−0.12^***^	0.10^***^	−0.15^***^	0.07^**^	0.22^***^	0.12^***^	0.25^***^	0.27^***^	–									
X10	−0.07^***^	0.16^***^	−0.20^***^	0.01	0.11^***^	0.02	0.26^***^	0.33^***^	0.41^***^	–								
X11	0.03	−0.02	0.11^***^	0.07^***^	0.03	0.02	−0.08^***^	−0.10^***^	0.01	−0.01	–							
X12	−0.04^*^	0.04^*^	0.02	0.04^*^	0.06^**^	0.08^***^	0.02	0.00	0.03	0.03	0.14^***^	–						
X13	−0.01	0.06^**^	−0.01	0.03	0.08^***^	0.08^***^	0.04^*^	0.02	0.05^*^	0.05^*^	0.13^***^	0.48^***^	–					
X14	−0.09^***^	0.10^***^	−0.06^***^	0.02	0.06^**^	0.06^**^	0.12^***^	0.14^***^	0.05^*^	0.09^***^	−0.01	0.01	0.05^*^	–				
X15	−0.06^**^	−0.03	0.08^***^	−0.08^***^	−0.06^**^	−0.10^***^	−0.05^*^	−0.16^***^	−0.07^***^	−0.05^*^	−0.01	−0.04^*^	−0.07^***^	−0.04	–			
X16	−0.01	−0.08^***^	0.12^***^	−0.08^***^	−0.07^***^	−0.11^***^	−0.07^***^	−0.16^***^	−0.08^***^	−0.08^***^	0.01	0.01	−0.02	−0.03	0.16^***^	–		
X17	−0.04^*^	0.04^*^	−0.04^*^	0.10^***^	0.14^***^	0.10^***^	0.00	0.03	0.04	0.04^*^	0.13^***^	0.29^***^	0.31^***^	0.05^*^	−0.11^***^	−0.05^*^	–	
X18	−0.01	0.04^*^	−0.24^***^	0.05^*^	0.01	0.03	0.04^*^	0.04^*^	0.00	0.03	0.08^***^	−0.06^***^	−0.07^***^	0.00	−0.06^**^	−0.07^***^	−0.02	–

*p ≤ 0.05, **p ≤ 0.01, and ***p ≤ 0.001.

**Table 3:** Multicollinearity diagnosis of factors associated with sexual abuse.

Variable	Tolerance	VIF
X1: Gender	0.88	1.14
X2:Sexual orientation	0.92	1.09
X3: Age (years)	0.82	1.23
X4: Condom use knowledge	0.91	1.10
X5: Condom use skills	0.85	1.18
X6: Condom use self-efficacy	0.87	1.15
X7: Marriage status	0.84	1.19
X8: Age at firs intercourse	0.77	1.31
X9: Number of partner	0.76	1.32
X10: Partner type	0.75	1.34
X11:Monthly expenditure (Yuan)	0.93	1.08
X12: Father’s education	0.74	1.35
X13: Mother’s education	0.73	1.38
X14: Family structure	0.96	1.05
X15: Family’s sexual education	0.93	1.08
X16: School’s sexual education	0.94	1.07
X17: Residential areas	0.84	1.19
X18: Region	0.91	1.09

### Ecological factors associated with child sexual abuse

After adjustment for potential confounding factors, monthly expenditure, number of partner and residential areas also lost their significance (Table 4). In the first model, both gender and sexual orientation were significantly associated with sexual abuse. More specifically, females (AOR = 1.81, 95% CI: 1.39–2.37) and sexual minority adolescents (AOR = 2.64, 95% CI: 2.01–3.48) were more likely to report sexual abuse. Therefore, based on gender and sexual orientation, four categories (i.e., heterosexual and sexual minority males and females) were created and then recoded into three dummy variables with heterosexual males as the reference category.

**Table 4:** Multivariable analysis of factors associated with child sexual abuse.

Variable	Model 1		Model 2	
	AOR	95% CI	AOR	95% CI
X1: Gender (0 = Male, 1 = Female)	1.81^***^	1.39–2.37		
X2: Sexual orientation (0 = Heterosexual, 1 = Sexual minority)	2.64^***^	2.01–3.48		
Gender and sexual orientation (Ref: Heterosexual male)				
Heterosexual female			1.86^***^	1.37–2.53
Sexual minority male			2.85^***^	1.74–4.68
Sexual minority female			4.77^***^	3.33–6.84
X3: Age (years) (1 = 15, 0 = 17)	1.84^**^	1.18–2.86	1.84^**^	1.18–2.86
X4: Condom use knowledge (0 = Low, 1 = High)	1.30^*^	1.02–1.66	1.30^*^	1.02–1.67
X5: Condom-use skills (0 = No, 1 = Yes)	1.38^*^	1.01–1.89	1.39^*^	1.01–1.90
X6: Condom-use self-efficacy (0 = Low, 1 = High)	1.35^*^	1.03–1.78	1.35^*^	1.03–1.78
X7: Marriage status (0 = Never-married, 1 = Ever-married)	2.18^***^	1.38–3.45	2.17^***^	1.37–3.44
X8: Age at firs intercourse (0 = Older than 14, 1 = Younger than 14)	8.62^***^	4.54–16.34	8.66^***^	4.56–16.45
X10: Partner type (0 = Stable, 1 = Casual)	2.64^**^	1.29–5.42	2.64^**^	1.29–5.42
X14: Family structure (0 = Intact families, 1 = Disrupted families)	1.57^***^	1.24–2.00	1.58^***^	1.24–2.01
X15:Family’s sexual education (0 = Ever, 1 = Never)	0.64^***^	0.49–0.82	0.64^***^	0.49–0.82
X16: School’s sexual education (Ref: Kindergarden or elementary school)				
Junior or senior middle school	0.67^***^	0.51–0.88	0.67^**^	0.51–0.88
College	0.42^***^	0.22–0.77	0.41^**^	0.22–0.77

*p ≤ 0.05, **p ≤ 0.01, and ***p ≤ 0.001.

At the individual level, as indicated in the second model, heterosexual females (AOR = 1.86, 95% CI: 1.37–2.53), sexual minority males (AOR = 2.85, 95% CI: 1.74–4.68) and females (AOR = 4.77, 95% CI: 3.33–6.84) were more likely to experience sexual abuse, when compared with heterosexual males. Furthermore, belonging to the younger age group (AOR = 1.84, 95% CI: 1.18–2.86), having more condom-use knowledge (AOR = 1.30, 95% CI: 1.02–1.67) and skills (AOR = 1.39, 95% CI: 1.01–1.90), and displaying higher self-efficacy for condom use (AOR = 1.35, 95% CI: 1.03–1.78) were significantly associated with increased risk of being sexually abused.

At the relationship and community level, adolescents from disrupted families (AOR = 1.58, 95% CI: 1.24–2.01) and those entering into a marriage (AOR = 2.17, 95% CI: 1.37–3.44), having casual sexual partners (AOR = 2.64, 95% CI: 1.29–5.42) and having first intercourse at a younger age (AOR = 8.66, 95% CI: 4.56–16.45) were more likely to report sexual abuse. On the contrary, those who had never discussed sex-related topics with their family members at home (AOR = 0.64, 95% CI: 0.49–0.82) were less likely to report sexual abuse than those who had ever discussed sex-related topics with their family members at home. Furthermore, adolescents who were offered sexuality education in middle schools (AOR = 0.67, 95% CI: 0.51–0.88) or in colleges (AOR = 0.41, 95% CI: 0.22–0.77) were less likely to report sexual abuse, compared to those who were offered sexuality education in kindergardens or elementary schools (see Table 4).

## Discussion

### Main findings of this study

Our results indicated that the overall prevalence of child sexual abuse was 13.0% for girls and 10.6% for boys, slightly lower than that (15.3% in girl and 13.8% in boys) estimated in a meta-analysis of data from 27 studies of 75,409 participants in China (19) and also lower than the international level (15). The relatively low child sexual abuse rates observed in this study might be due to many factors such as inhibited disclosure, social desirability, conformity with conventional moral values, smaller and more intact families, based on the analysis conducted by Finkelhor and colleagues (32). Furthermore, this study identified the effects of six individual factors (i.e., gender, sexual orientation, age, knowledge, skills and self-efficacy regarding condom use) and six relationship and community-related variables (i.e., marriage status, partner type, age at firs intercourse, family structure, family-based and school-based sex education) on the history of child sexual abuse. Some of these findings were consistent with previous research.

### Comparison with other studies

#### Gender and sexual orientation

The emergence of gender as having a significant association with the exposure to child sexual abuse was consistent with previous findings (15, 19) in which girls were significantly more likely than boys to report sexual abuse experience. This finding might be explained by three factors (15). First, men show greater sexual desire (41), are more aggressive (41) and are more likely to commit acts of sexual violence (42), and thus girls are more likely to become victims of sexual abuse (43). For example, a survey based on 1,002 cases of child sexual abuse (43) indicated that virtually all perpetrators were male, and more than 80% of the victims were girls. Second, boys were traditionally regarded as potential aggressors rather than as potential victims. Therefore, boys might be more reluctant to reveal their sexual abuse experience for fear of being labeled as an inadequate, feminine-like male, the instigator of the sexual abuse, or a homosexual (44). Third, consistent with Mennicke and colleagues (38), sexual minority girls had the highest rate of sexual abuse (30.0%), followed by sexual minority boys (26.4%) and heterosexual girls (9.6%), then heterosexual boys (8.4%). Sexual minority boys were more likely to be sexually abused than heterosexual girls. However, due to the fact that the majority of boys are heterosexual, girls are the most victimized group.

Consistent with earlier findings (3, 38, 39), sexual minority adolescents were more likely to experience sexual abuse than heterosexual adolescents. These disparities might be explained by sexual minority stressors (38) such as internalized homophobia, stigma consciousness, openness about sexual orientation, and discrimination. In addition, Murchison et al. (45) confirmed that minority stress, specifically internalized homophobia, predicted unwanted sexual experiences among LGBQ undergraduates.

#### Age

Younger adolescents (i.e., 15 years) were more likely to report sexual abuse than older adolescents (i.e., 17 years). This phenomenon might be explained by the fact that discussions about sexual abuse among younger adolescents have become less taboo in recent years, and therefore younger adolescents were more knowledgeable of the definition of broad sexual abuse and were more willing to disclose sexual abuse experience in a strongly inhibited culture (19, 32).

#### Sexuality knowledge and sex education

In this study, 15- to 17-years-old adolescents who had received sexuality education were more likely to report sexual abuse experience. One possible explanation for this phenomenon was that adolescents who had been sexually abused might have more questions regarding such experience, and thus were more likely to talk to their family members or their teachers regarding sex issues and be educated about the advantages of condom use and preventive measures such as condom use skills, condom negotiation, and sex refusal skills. The other possible explanation is that China is still a relatively traditional and conservative society, in which sex is often considered a taboo subject and most Chinese people feel embarrassed to talk openly about it. Therefore, sexuality education program, despite being introduced into schools more than three decades ago, are widely considered to be inadequate and even unevenly implemented because of conservative attitudes towards sexuality, insufficient class hours, lack of teachers and shortage of teaching resources (46, 47). Furthermore, Li and colleagues (47) proposed the idea that Chinese people were subject to long-term sexual repression and could be more curious to experience sex once they learn something about sex. In addition, parent-adolescent sex communication was found to be significantly associated with adolescent sexual initiation (29). Consistent with the findings of Houck et al. (28), early initiation of sex and subsequent sexual risk behaviors (e.g., casual sex) in this study were also found to be significantly associated with the history of sexual abuse. Therefore, it is hypothesized that sex education might expose adolescents to the risk of being sexually abused via a variety of mechanisms such as early initiation of sex and casual sex.

#### Relationship

In agreement with Priebe and Svedin (2), students with the history of sexual abuse were more likely to come from disrupted family. This would be due to the fact that adolescents not living with their biological parents had lower perception of parental warmth and experienced lower levels of parental monitoring, thus contributing to the formation of anxiety and loneliness and increasing their odds of being exposed to sexual abuse from opportunistic perpetrators. Furthermore, perpetrators of sexual abuse might be their stepparents (48), intimate partners (27, 49), sexual partners (28), or spouses (30). For example, Alexandre et al. (50) found that children living at home with their mother and stepfather were twice more likely to be victims of physical abuse compared to those living with their genetic parents. In addition, a history of sexual abuse was found to be significantly associated with the existence of risky sexual behaviors such as early sexual debut (2, 49), unprotected sex (28, 49) and multiple sexual partners (51). Consistent with our hypothesis, coming from disrupted families, entering into a marriage, having casual sexual partners and having their first intercourse at a younger age were found to increase the risk of being sexually abused.

#### Community

According to Ji and colleagues (19), rural children, especially left-behind children in rural China (52) were more likely to experience sexual abuse than their urban counterparts due to the migration of their fathers or both parents into cities. Furthermore, they found lower rates of sexual abuse for children in non-mainland provinces such as Hong Kong and Taiwan (19), contrary to the findings of Chan’s 2012 study (53). And they also reasoned that methodological factors (e.g., Chen’s multi-question questionnaire was used primarily in mainland studies), rather than the Westernized culture, contributed to the discrepancy between their and Chan’s findings. However, after restricting our sample to adolescents in mainland China and adjusting the effects of living arrangements, there were no significant associations existing between child sexual abuse and geographical characteristics and area of residence.

### Limitations

Several limitations must be taken into consideration in interpreting the findings. First, due to the cross-sectional nature of our data, the causal direction of these effects remains uncertain. For example, significant association between risky sexual behaviors and the history of child sexual abuse does not necessarily mean that the former caused the latter. It can also mean that child sexual abuse itself contributed to the persistence of risky sexual behaviors (54). Therefore, it also remains unclear the intervention recommendations based on the above research findings, which will be described in more detail in later sections, can play a significant role in preventing child sexual abuse. Second, the history of sexual abuse was assessed by only one item that vaguely asked participants whether or not they had ever experienced sexual abuse, which did not define what experiences or behaviors constitute sexual abuse. Therefore, the answers to this question may be heavily influenced by the participants’ subjective perceptions and definitions of sexual abuse. Third, a series of variables such as the nature, time, location and frequency of sexual abuse, the perpetrator-victim relationship and social norms were not obtained in this study and therefore could not be assessed as possible predictors for sexual abuse. Fourth, non-probability sampling methods were applied to recruit respondents in this study due to limited resources and time, and the generalizability of the findings would be limited due to the lack of representativeness. Fifth, the respondents were only asked to indicate whether they communicated with their family members regarding sex-related issues or when they received school-based sex education. However, no discussion topics or the content of sex education information were not assessed in this study. Sixth, sexual minorities are a heterogeneous group composed of lesbians, gay men, bisexual men, bisexual women, male-to-female (MtF) and female-to-male (FtM) transgender (LGBT) individuals. Due to the small sample size, all LGBT adolescents (*n* = 479) were categorized as sexual minority adolescents. Finally, the data were obtained by self-report and might be subject to recall and social desirability bias.

### Implications of the study

In spite of the above-mentioned limitations, the findings from our study have several implications for the design and implementation of child sexual abuse prevention programs. To the best of our knowledge, ours is the first to determine the prevalence of child sexual abuse among a large, diverse sample of adolescents selected from the whole country and simultaneously identify its associated factors using the ecological model. Our findings suggested that more than one tenth of adolescents reported experiencing sexual abuse. Multivariate Logistic regression analyses further demonstrated that adolescents with a history of sexual abuse were more likely to be females, sexual minority identity, belong to the younger age group, have higher levels of knowledge, skills and self-efficacy regarding condom use, enter into a marriage, having casual sexual partners, and have first intercourse at a younger age, come from disrupted families, discuss sex-related topics with their family members at home, and receive school-based sexuality education earlier. Therefore, three-levels of prevention programs and strategies are recommended to prevent child sexual abuse in China.

1. Targeting adolescents with high-risk characteristics. Females, younger adolescents, sexual minority adolescents and those from disrupted families and those who have ever been married, having casual sexual partners, have first intercourse at a younger age had a higher tendency to being sexually abused and were classified as high-risk individuals and should therefore be prioritized for future intervention efforts.
2. Educating young children and their parents about child sexual abuse prevention. Our study suggests that parent-adolescent communication on sexual and reproductive health issues was very limited (22.3%) in mainland China, and the effect of this communication is questionable by the comparison of the rates of sexual abuse for adolescents who had ever or never discussed sex-related topics with their parents at home. Therefore, parents and children should be educated on the dangers of child sexual abuse and encouraged to report cases not only to serve as a deterrent to abusers but to help the victims to receive comprehensive care such as emergency medical care, counselling, collecting forensic evidence and legal support (12).
3. Optimizing the involvement of parents, school, community, society and government in sexuality education. Sexuality education is a lifelong process. It includes, but is not limited to anatomy, physiology, pregnancy, reproduction, sexual development, sexual orientation, gender, healthy relationships, and personal safety. Adolescents can learn about sexuality from their parents, peers, teachers, community and society. Furthermore, according to the newly revised Law on the Protection of Minors, which took effect on June 1, 2021, the schools, families, the public security should cooperate with relevant departments to protect children from sexual abuse.

## Data availability statement

All data generated or analyzed during this study are included in this published article. The raw data supporting the conclusions of this article will be made available by the corresponding author upon reasonable request.

## Ethics statement

The studies involving humans were approved by the academic ethics and moral supervision committee of Hubei University of Science and Technology. The studies were conducted in accordance with the local legislation and institutional requirements. Written informed consent for participation in this study was provided by the participants' legal guardians/next of kin.

## Author contributions

JW, GF, YX, and CY wrote the main manuscript text. MP, ZZhan, HZ, YZ, LL, ZY, JL, FL, DL, SW, and BZ prepared Tables 1–4 and Figure 1. XL, MH, JC, ZL, GY, ZZhao, YL, and FR collected and analyzed the data. All authors contributed to the article and approved the submitted version.
